# Combined impact of healthy lifestyle factors on colorectal cancer: a large European cohort study

**DOI:** 10.1186/s12916-014-0168-4

**Published:** 2014-10-10

**Authors:** Krasimira Aleksandrova, Tobias Pischon, Mazda Jenab, H Bas Bueno-de-Mesquita, Veronika Fedirko, Teresa Norat, Dora Romaguera, Sven Knüppel, Marie-Christine Boutron-Ruault, Laure Dossus, Laureen Dartois, Rudolf Kaaks, Kuanrong Li, Anne Tjønneland, Kim Overvad, José Ramón Quirós, Genevieve Buckland, María José Sánchez, Miren Dorronsoro, Maria-Dolores Chirlaque, Aurelio Barricarte, Kay-Tee Khaw, Nicholas J Wareham, Kathryn E Bradbury, Antonia Trichopoulou, Pagona Lagiou, Dimitrios Trichopoulos, Domenico Palli, Vittorio Krogh, Rosario Tumino, Alessio Naccarati, Salvatore Panico, Peter D Siersema, Petra HM Peeters, Ingrid Ljuslinder, Ingegerd Johansson, Ulrika Ericson, Bodil Ohlsson, Elisabete Weiderpass, Guri Skeie, Kristin Benjaminsen Borch, Sabina Rinaldi, Isabelle Romieu, Joyce Kong, Marc J Gunter, Heather A Ward, Elio Riboli, Heiner Boeing

**Affiliations:** Department of Epidemiology, German Institute of Human Nutrition Potsdam-Rehbrücke, Nuthetal, Germany; Molecular Epidemiology Group, Max Delbrueck Center for Molecular Medicine (MDC), Berlin-Buch, Germany; International Agency for Research on Cancer (IARC-WHO), Lyon, France; National Institute for Public Health and the Environment (RIVM), Bilthoven, Netherlands; Department of Gastroenterology and Hepatology, University Medical Center, Utrecht, the Netherlands; Department of Epidemiology and Biostatistics, School of Public Health, Imperial College London, London, UK; Department of Epidemiology, Rollins School of Public Health, Winship Cancer Institute, Emory University, Atlanta, GA USA; Instituto de Investigacion Sanitaria de Palma (IdISPa), Hospital Universitario Son Espases, Palma de Mallorca, Spain; CIBER Fisiopatología de la Obesidad y Nutrición (CIBEROBN), Santiago de Compostela, Spain; Inserm, Centre for research in Epidemiology and Population Health (CESP), U1018, Nutrition, Hormones and Women’s Health team, F-94805 Villejuif, France; Univ Paris Sud, UMRS 1018, F-94805 Villejuif, France; IGR, F-94805 Villejuif, France; Division of Cancer Epidemiology, German Cancer Research Centre, Heidelberg, Germany; Diet, Genes and Environment Danish Cancer Society Research Center, Copenhagen, Denmark; Department of Public Health, Section for Epidemiology, Aarhus University, Aarhus, Denmark; Public Health Directorate, Asturias, Spain; Unit of Nutrition, Environment and Cancer, Cancer Epidemiology Research Programme, Catalan Institute of Oncology (ICO-IDIBELL), Barcelona, Spain; Escuela Andaluza de Salud Pública. Instituto de Investigación Biosanitaria de Granada (Granada.ibs), Granada, Spain; CIBER de Epidemiología y Salud Pública (CIBERESP), Madrid, Spain; Epidemiology and Health Information, Public Health Division of Gipuzkoa, Basque Regional Health Department, San Sebastian, Spain; Department of Epidemiology, Murcia Regional Health Authority, Murcia, Spain; Navarre Public Health Institute, Pamplona, Spain; Clinical Gerontology Unit, Addenbrooke’s Hospital, University of Cambridge School of Clinical Medicine, Cambridge, UK; MRC Epidemiology Unit, Institute of Metabolic Science, University of Cambridge School of Clinical Medicine, Cambridge, UK; Cancer Epidemiology Unit, Nuffield Department of Population Health, University of Oxford, Oxford, UK; Hellenic Health Foundation, Athens, Greece; Bureau of Epidemiologic Research, Academy of Athens, Athens, Greece; Department of Hygiene, Epidemiology and Medical Statistics, University of Athens Medical School, Athens, Greece; Department of Epidemiology, Harvard School of Public Health, Boston, MA USA; Molecular and Nutritional Epidemiology Unit, Cancer Research and Prevention Institute – ISPO, Florence, Italy; Epidemiology and Prevention Unit, Fondazione IRCCS Istituto Nazionale dei Tumori, Milan, Italy; Cancer Registry and Histopathology Unit, “M.P.Arezzo” Hospital, Ragusa, Italy; HuGeF - Human Genetics Foundation – Torino, Molecular and Genetic Epidemiology Unit, Turin, Italy; Department of clinical and experimental medicine-Federico II University, Naples, Italy; Department of Epidemiology, Julius Center for Health Sciences and Primary Care, University Medical Center, Utrecht, the Netherlands; Department of Radiation Sciences, Oncology, Umeå University, Umeå, Sweden; Department of Odontology, Umeå University, Umeå, Sweden; Diabetes and Cardiovascular Disease, Genetic Epidemiology, Department of Clinical Sciences in Malmö, Lund University, Malmö, Sweden; Department of Clinical Sciences, Division of Internal Medicine, Skåne University Hospital Malmö, Lund University, Malmö, Sweden; Department of Community Medicine, Faculty of Health Sciences, University of Tromso, The Arctic University of Norway, Tromsø, Norway; Department of Research, Cancer Registry of Norway, Oslo, Norway; Department of Medical Epidemiology and Biostatistics, Karolinska Institutet, Stockholm, Sweden; Samfundet Folkhälsan, Helsinki, Finland

**Keywords:** lifestyle factors, combined impact, population attributable risks, colorectal cancer, European Prospective Investigation into Cancer and Nutrition (EPIC)

## Abstract

**Background:**

Excess body weight, physical activity, smoking, alcohol consumption and certain dietary factors are individually related to colorectal cancer (CRC) risk; however, little is known about their joint effects. The aim of this study was to develop a healthy lifestyle index (HLI) composed of five potentially modifiable lifestyle factors – healthy weight, physical activity, non-smoking, limited alcohol consumption and a healthy diet, and to explore the association of this index with CRC incidence using data collected within the European Prospective Investigation into Cancer and Nutrition (EPIC) cohort.

**Methods:**

In the EPIC cohort, a total of 347,237 men and women, 25- to 70-years old, provided dietary and lifestyle information at study baseline (1992 to 2000). Over a median follow-up time of 12 years, 3,759 incident CRC cases were identified. The association between a HLI and CRC risk was evaluated using Cox proportional hazards regression models and population attributable risks (PARs) have been calculated.

**Results:**

After accounting for study centre, age, sex and education, compared with 0 or 1 healthy lifestyle factors, the hazard ratio (HR) for CRC was 0.87 (95% confidence interval (CI): 0.44 to 0.77) for two factors, 0.79 (95% CI: 0.70 to 0.89) for three factors, 0.66 (95% CI: 0.58 to 0.75) for four factors and 0.63 (95% CI: 0.54 to 0.74) for five factors; *P*-trend <0.0001. The associations were present for both colon and rectal cancers, HRs, 0.61 (95% CI: 0.50 to 0.74; *P* for trend <0.0001) for colon cancer and 0.68 (95% CI: 0.53 to 0.88; *P*-trend <0.0001) for rectal cancer, respectively (*P*-difference by cancer sub-site = 0.10). Overall, 16% of the new CRC cases (22% in men and 11% in women) were attributable to not adhering to a combination of all five healthy lifestyle behaviours included in the index.

**Conclusions:**

Combined lifestyle factors are associated with a lower incidence of CRC in European populations characterized by western lifestyles. Prevention strategies considering complex targeting of multiple lifestyle factors may provide practical means for improved CRC prevention.

**Electronic supplementary material:**

The online version of this article (doi:10.1186/s12916-014-0168-4) contains supplementary material, which is available to authorized users.

## Background

Colorectal cancer (CRC) is the third most common cancer in men (746,000 cases per year, 10.0% of the total cancer incidence) and the second in women (614,000 cases per year, 9.2% of the total cancer incidence) worldwide [1]. There is a wide geographical variation in CRC incidence rates across the world with almost 55% of the cases occurring in more developed regions [1]. The parallel between the cancer frequency rates and the level of ‘westernisation’ points to an important role of lifestyle factors in the etiology of CRC [2-13]. In support of this hypothesis, the World Cancer Research Fund/American Institute for Cancer Research (WCRF/AICR) expert panel acknowledged that high physical activity and high intakes of dietary fibre, fish, nuts, dairy products, and fruits and vegetables are associated with a lower CRC risk, whereas high body mass index (BMI) and waist circumference, smoking, alcohol consumption, and red and processed meat intakes are related to a higher CRC risk [14-16]. While individual roles of these lifestyle factors have been extensively investigated, little is known about their joint effects. Most epidemiological studies explored individual health behaviours by treating other lifestyle factors as covariates in statistical models; however, in real life it is uncommon that people practice isolated behaviours. A multidimensional lifestyle approach would be more informative for exploring disease etiology, as well as for translating epidemiological findings into meaningful prevention strategies. Furthermore, estimation of health impact measures, such as population attributable risks (PARs), may provide better means for public health decision making, because PARs address what proportion of disease risk may be prevented over a specified time interval if a risk factor (or a combination of risk factors) is absent in a given population [17]. In addition, differences between colon and rectal anatomical cancer subtypes and sex have been previously shown to exist for associations with several lifestyle factors, such as excess body weight, waist circumference and physical inactivity [18-20]; however, it is not clear whether such differences may be valid also for combinations of factors. Finally, varying combinations of risk factors differentially contribute to diabetes, cardiovascular diseases and cancer overall [21]; therefore, it may be important to investigate specific lifestyle patterns in relation to CRC risk. To address these aspects, we aimed to develop a healthy lifestyle index (HLI) composed of five potentially modifiable lifestyle factors – healthy weight, physical activity, non-smoking, limited alcohol consumption and a healthy diet – and to explore the association of this index with CRC incidence using data collected within the European Prospective Investigation into Cancer and Nutrition (EPIC) cohort. Furthermore, we aimed to evaluate the combined impact of these lifestyle factors in terms of PARs overall and according to colon and rectal cancer anatomical sub-site and by sex.

## Methods

### Study design and population

A total of 521,330 men and women, 25- to 70-years old, were recruited between 1 January 1992 and 31 December 2000 from 23 centres in 10 European countries: Denmark, France, Germany, Greece, Italy, the Netherlands, Norway, Spain, Sweden and the United Kingdom. Approval for the EPIC study was obtained from the ethical review boards of the International Agency for Research on Cancer and from all local institutions where subjects had been recruited for the EPIC study [see Additional file 1: Table S1]. Written informed consent was obtained from all participants before joining the EPIC study. Details of the recruitment and study design have been published elsewhere [22]. We excluded participants with missing data on dietary factors (n = 6,193), waist circumference measurements (n = 109,302), smoking history (11,746), physical activity (n = 69,393), underweight participants (BMI <18; n = 95,381) and participants with prevalent diabetes reported at study baseline (n = 13,049). Due to missing data on waist circumference measurements, participants from Norway (n = 35,890) were excluded from the analyses. Consequently, the study population for the current analyses was comprised of 3,759 CRC cases (2,369 colon cancers and 1,390 rectal cancers) and 343,478 non-cases.

### Case ascertainment

Cancer cases were identified through population cancer registries in Denmark, Italy, the Netherlands, Spain, Sweden and the United Kingdom. In France, Germany and Greece, a combination of methods was used including health insurance records, cancer pathology registries and active follow-up of study participants and their next of kin. Follow-up began at the date of enrolment and ended at the date of diagnosis of cancer, death or last complete follow-up. The last update of endpoint information was done up to 31 September 2010. Cancer incidence data were coded according to the 10th revision of the International Statistical Classification of Diseases, Injuries and Causes of Death [23] and the second revision of the International Classification of Diseases for Oncology [24]. Only the first primary neoplasm was included in the analysis; non-melanoma skin cancer was excluded.

### Assessment of lifestyle factors

At baseline, participants filled out extensive medical, dietary and lifestyle questionnaires, including questions on alcohol use, smoking status, physical activity, education and previous illnesses. Body weight, height and waist circumference were measured in all centres except for EPIC-Oxford (health-conscious population) and France where anthropometric measurements were self-reported [22]. Usual food intakes were measured by using country-specific validated dietary questionnaires, and individual nutrient intakes were derived from foods included in the dietary questionnaires through the standardised EPIC Nutrient Database [25]. All dietary variables used in the present study were calibrated by using an additive calibration method as previously described [26].

### HLI definition

We generated the HLI based on *a priori* knowledge of the CRC risk factors [2-13] and available national and international public health recommendations (that is, WCRF/AICR (2007)) [15,16]. We used a binary score for each factor in order to allow easy translation of findings into a prevention practice (Table 1). Participants were assigned one point for each of the following behaviours assessed at study baseline: healthy weight (BMI <25 [27] or waist circumference <80 cm for women and <94 cm for men [28]); not smoking or former smoking, high physical activity [13], adherent to alcohol consumption recommendations of the WCRF/AICR (2007) [16] and having a healthy diet. Healthy diet was evaluated based on a dietary quality index including eight dietary factors (fruits, vegetables, red and processed meat, fibre, fish, nuts, garlic and yogurt), which were previously shown to be related to CRC [see Additional file 2: Table S2]. Finally, the HLI was constructed by summing the binary score for each of the five lifestyle factors which ranged from 0 (least healthy) to 5 (most healthy) points.

**Table 1:** **Description and prevalences of the factors comprising the Healthy Lifestyle Index (HLI), the EPIC Cohort (1992 to 2010)**

**Lifestyle factor**	**Index points**	**Description**	**Prevalence in the EPIC study population (%)**		
			**Men**	**Women**	**Overall**
**Overweight and obesity** ^**a**^	0	Overweight or obese: BMI ≥25 kg/m^2^ or waist circumference ≥94 cm for men and ≥80 cm for women			
	1	Healthy weight: BMI 18 to 25 kg/m^2^ or waist circumference <94 for men cm and <80 for women	52.2	62.1	58.6
**Physical activity** ^**b**^	0	Low and very low physical activity: sedentary or standing occupation and recreational METs ≤57 for men and METs ≤82 for women			
	1	High and very high physical activity: manual or heavy manual occupation and recreational METs >57 for men and METs >82 for women	50.3	52.6	51.7
**Smoking**	0	Smoking: current smokers			
	1	Non-smoking: never or former smokers	69.1	79.8	76.1
**Alcohol consumption**	0	Heavy alcohol consumption: not adherent to alcohol consumption recommendations of WCRF/AICR (2007) [15] for two standard drinks a day (>24 g/day) for men and one standard drink a day (>12 g/day) for women			
	1	Limited alcohol consumption: adherent to alcohol consumption recommendations of WCRF/AICR (2007) [15,16] for two standard drinks a day (≤24 g/day) for men and one standard drink a day (≤12 g/day) for women	66.0	75.9	72.4
**Diet quality** ^**c**^	0	Unhealthy diet quality: 0 to 4 points of the diet index of colorectal cancer related foods			
	1	Healthy diet quality: 5 to 8 points of the diet index of colorectal cancer related foods	60.9	59.6	60.1

^a^Based on the World Health Organisation’s standard cutoff point for overweight [27] or waist circumference <80 cm for women and <94 cm for men according to the European Group for the Study of Insulin Resistance (EGIR) recommendations for European populations [28]. ^b^A MET is defined as the ratio of work metabolic rate to a standard metabolic rate of 1.0 (4.184 kJ) kg^−1^ h^−1^; 1 MET is considered a resting metabolic rate obtained during quiet sitting. The MET values assigned to the non-occupational data were 3.0 for walking, 6.0 for cycling, 4.0 for gardening, 6.0 for sports, 4.5 for home repair (do-it-yourself work), 3.0 for housework and 8.0 for stair climbing [13]. ^c^Healthy diet was evaluated based on a dietary quality index including eight dietary factors (fruits, vegetables, red and processed meat, fibre, fish, nuts, garlic and yogurt), which were previously shown to be related to CRC overall and in the EPIC data [2-8,11,12,38-40] (Additional file 2, Table S2). BMI, body mass index (calculated as weight in kilograms divided by height in squared metres); EPIC, European Prospective Investigation into Cancer and Nutrition; METs , metabolic equivalents of energy expenditure (MET)-hours per week per year; WCRF/AICR, World Cancer Research Fund/American Institute for Cancer Research.

### Statistical analysis

In descriptive analyses, we estimated the prevalence of each individual lifestyle factor included in the HLI and examined the baseline characteristics of the study participants according to an increasing HLI score. We next evaluated the association of the lifestyle factors modeled individually and in combination - as an index variable (HLI) - with risk of CRC. We used multivariable Cox proportional hazards models to calculate hazard ratios and 95% confidence intervals (CIs). Age (continuous) was used as the primary time-dependent variable in all models, with entry time defined as the subject’s age at recruitment (years) and exit time as the age at diagnosis, death or return of the last follow-up questionnaire, whichever came first. Individual associations of the lifestyle factors with CRC were evaluated with each lifestyle factor modeled as a binary variable. The base model was stratified by EPIC study centre, and adjusted for age at study recruitment, sex (in the sex combined model) and educational level. The multivariable model for individual lifestyle factors was additionally adjusted for the remaining lifestyle factors. In these analyses, participants with 0 points (least healthy) were the reference group. To evaluate the association of the lifestyle factors in combination, we modeled the HLI both as an ordinal variable and as a categorical variable according to six categories (0 to 5 points) with the least healthy group (0 points) as the reference group. *P*-value for the linear trend was calculated using the Wald test treating the index as a continuous variable. Since normal body weight may be considered as a consequence of healthy lifestyle behaviours (that is, high physical activity and a healthy diet), we performed a subgroup analysis excluding participants with healthy weights (0 to 4 scores). In order to test whether individual factors may statistically explain the association between the combined index and CRC, we added each of the factors to the multivariable-adjusted model one at a time. The percent change in the regression coefficient with adjustment for each individual lifestyle factor was compared with the multivariable model. The corresponding 95% CI was calculated based on Fieller’s theorem [29]. In addition, we examined the multivariable risks of CRC according to all possible combinations of lifestyle factors. The five dichotomised healthy lifestyle factors yielded thirty two combinations and the hazard ratios (HRs) for each of these combinations were calculated using participants who had no healthy factors as the reference group. All analyses were performed separately for colon and rectal cancer and by sex. Differences by cancer site were tested by competing risk analyses using the model of Lunn-McNeil [30], whereas the differences by sex were tested based on the likelihood ratio test by generating cross-product terms in multivariable models. Under the assumption that the associations are causal, we calculated the percentage of PARs and 95% CIs to estimate the proportion of CRC cases attributed to each individual lifestyle factor, as well as to lack of adherence to all of the five healthy lifestyle factors. For these analyses, we compared participants in the high-risk category with the rest of the population for each factor and for the index. PARs for single lifestyle factors were derived from equations by Miettinen [31] taking the strata specific prevalences of cases and multivariable-adjusted HRs into consideration. Attributable risks for factors in combination (PAR_j_) were determined using an equation by Bruzzi *et al*. [32]:

$$ PA{R}_{\mathrm{j}}={\rho}_{\mathrm{j}}\frac{{\mathrm{RR}}_{\mathrm{J}}\hbox{-} 1}{\mathrm{RR}\mathrm{j}}, $$

where ρ_j_ is the prevalence of individuals not in the low risk group and RR_j_ is the associated multivariable-adjusted hazard ratio. Upper and lower CIs of the PARs were calculated based on the formula by Whittemore *et al*. [33], as reported in previous analyses [34-36]. We stratified the analysis according to median age (52.4 years) and country in order to examine the potential of effect modification by any of these factors. In addition, we performed sensitivity analysis to account for possible influence on the associations of family history as an established risk factor for CRC using available data from the EPIC centres in France, Spain and the United Kingdom, where 5,309 participants have reported having a family history of CRC. We also performed analyses comparing participants with and without missing data on major lifestyle exposure variables in order to control for potential missing data bias. Finally, we performed a lag analysis excluding participants diagnosed with cancer within the first two years of study follow-up to control for potential influence of sub-clinical disease on these associations. All statistical analyses were performed using the Statistical Analysis System (SAS) (version 9.2), Enterprise Guide User Interface (version 4.3); SAS Institute, Inc., Cary, NC, USA. All *P* values were based on two-sided tests, and *P* < .05 was considered statistically significant.

## Results and discussion

**Figure 1:**
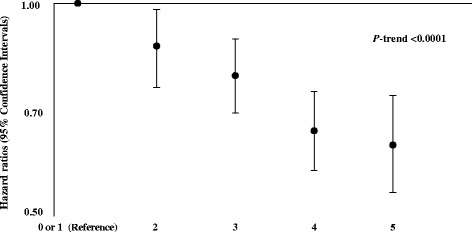
**Multivariable-adjusted hazard ratios (95% confidence intervals) of colorectal cancer according to increasing number of healthy lifestyle factors.** Healthy lifestyle index (range 0 to 5 points) is calculated by summing the binary lifestyle factor variables (0, 1) including overweight and obesity, physical activity, smoking, alcohol consumption and diet quality. Participants received 1 point if they had any of the following behaviours: healthy weight, physically active, non-smokers or former smokers, limited alcohol consumption or healthy diet quality. The hazard ratios are calculated after stratification by EPIC study centre and multivariable adjustment for age at study recruitment, sex and educational status (none, primary school, technical/professional school/not specified). *P*-value for the linear trend was calculated using the Wald test treating the index as a continuous variable. EPIC. European Prospective Investigation into Cancer and Nutrition.

**Figure 2:**
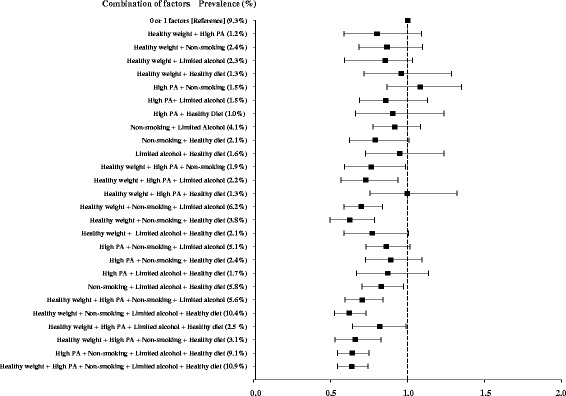
**Multivariable-adjusted hazard ratios of colorectal cancer according to combinations of healthy lifestyle factors.** HRs are shown for persons with the respective combination of healthy lifestyle factors compared with persons with none or one of the lifestyle factors; The multivariable model is stratified by EPIC study centre and adjusted for age at study recruitment, sex and educational status (none, primary school, technical/professional school/ not specified). The presented prevalences (%) represent the frequency distribution of the respective combinations of healthy lifestyle factors among the total study population. Within the reference group of individuals with 0 or 1 healthy lifestyle factors, 0.8% had 0 factors, 1.1% had only healthy weight; 0.85% had only high physical activity, 1.6% had only non-smoking, 1.4% had only limited alcohol and 0.9% had only healthy diet. CI, confidence interval; EPIC, European Prospective Investigation into Cancer and Nutrition; HR, hazard ratio.

The median follow-up time of the study was 12 years (5th to 95th centile: 7.0 to 14.5). The total cohort’s median age was 51.8 ± 10.2 years, and 121,116 (35%) of the participants were men. Among the study population, 203,595 (59%) participants had BMI and waist circumference within the recommended range, 179,787 (52%) had high physical activity, 264,153 (76%) were non-smokers (among these, 63% had never smoked and 37% were former smokers), 251,523 (72%) had alcohol intake within the recommended limits, and 208,562 (60%) had a healthy diet as assessed by the dietary quality index (Table 1). The participants having a higher HLI were more likely to be women and tended to have a higher educational level (Table 2). Each healthy lifestyle factor was associated with a reduction in CRC risk after taking age, sex, educational status and the remaining lifestyle factors into account (Table 3). Compared with participants with no or one healthy lifestyle factors, the multivariable-adjusted HR for CRC was 0.87 (95% CI: 0.76 to 0.98) for two factors, 0.79 (95% CI: 0.70 to 0.89) for three factors, 0.66 (95% CI: 0.58 to 0.75) for four factors and 0.63 (95% CI: 0.54 to 0.74) for five factors; *P*-trend <0.0001 (Figure 1). When evaluated ordinally, each additional healthy lifestyle factor was associated with a 12% lower risk of CRC (HR *for a one point increase on the index* = 0.88; 95% CI: 0.86 to 0.92), 13% lower risk of colon cancer (HR = 0.87; 95% CI = 0.83 to 0.90) and 9% lower risk of rectal cancer (HR = 0.91; 95% CI: 0.87 to 0.95; *P*-difference by cancer sub-site = 0.10; Table 4). Overall, the associations between HLI and CRC were stronger in men compared to women (*P*-interaction = 0.03); however, when stratified by cancer site it became obvious that these differences could be mostly observed for rectal cancer but not for colon cancer (*P*-interaction = 0.0008). Additional adjustment for each of the individual lifestyle factors did not materially change the associations of HLI with CRC [see Additional file 3: Table S3]. However, in analyses by cancer site and sex, overweight and obesity appeared to statistically significantly explain the association of HLI with colon cancer in men by 29% (95% CI: 7% to 62%). In analysis based on an index that excluded healthy weight, the associations remained similar (that is, HR *for a one point increase on the index =* 0.89; 95% CI: 0.87 to 0.92, for CRC). The estimated PARs of CRC representing the percentage of the population attributable to non-adherence to the particular healthy lifestyle behaviour were 8%, 3%, 4%, 4% and 5% for healthy weight, physical activity, non-smoking, limited alcohol consumption and a healthy diet, respectively. Overall 16% of the new CRC cases (22% in men and 11% in women) were attributable to not adhering to a combination of all of these five healthy lifestyle behaviours (Table 5). The results revealed a cancer-site and sex-specific gradient in estimated PARs such that 36% of rectal cancer cases in men and 20% of colon cancer cases in women were attributable to not adhering to all five healthy lifestyle factors, while no significant PARs were seen for colon cancer in men and rectal cancer in women. When we conducted analyses according to different combinations of two, three and four healthy lifestylefactors relative to no or one factors, we did not observe a lower risk for any of the combinations of two factors; whereas the risk of CRC was lower for several combinations of three healthy lifestyle factors (Figure 2). Among these, the combination of healthy weight, non-smoking and a healthy diet (HR = 0.62; 95% CI: 0.49 to 0.78) was associated with as lower risk as the combination of five lifestyle factors (HR = 0.63; 95% CI: 0.54 to 0.74). For most combinations, HLI scores of four and five were similarly protective. In stratified analyses, no substantial differences in the results were seen according to the age strata of less or more than 52.4 years (*P*-difference = 0.49) and by EPIC participating country [see Additional file 4: Figure S1; *P*-difference = 0.17]. Overall there have not been major differences between participants with and without missing data according to the main study characteristics and exposure variables (data not shown). In sensitivity analyses, in a multivariable-adjusted model including age, sex and education, additional adjustment for family history did not substantially alter the risk estimate for the association between HLI and CRC: HR = 0.88 (95%CI: 0.86 to 0.91); *P*-value <0.0001. The results were also not markedly changed after excluding cases diagnosed with CRC within the first two years of study follow-up; the HR for one point increase on the index was 0.77 (95% CI: 0.71 to 0.83).

**Table 2:** **Baseline characteristics of participants by Healthy Lifestyle Index (HLI) score, the EPIC cohort (1992 to 2010)**

**Characteristics**	**Healthy lifestyle index points**					
	**0**	**1**	**2**	**3**	**4**	**5**
Participants, number (%)	2,783 (0.8)	20,865 (6.0)	66,110 (19.0)	113,171 (32.6)	106,518 (30.7)	37,790 (10.9)
Colon cancer, number of cases	28	188	526	816	602	209
Rectal cancer, number of cases	12	121	326	459	348	124
Colorectal cancer, number of cases	40	309	852	1275	950	333
***Socio-demographic characteristics:***						
Age, mean, SD	52.7	52.1	52.2	51.9	51.5	50.8
Men,%	58.5	51.0	42.9	34.5	28.3	29.6
University degree,%	21.4	22.8	22.9	23.6	24.1	25.5
*Lifestyle factors:*						
BMI, kg/m^2^median	28.1	27.4	26.7	25.6	24.5	23.5
Waist circumference, cm, median						
*Men*	101.0	100.0	98.0	95.0	90.5	87.3
*Women*	89.0	85.0	83.0	80.0	77.0	73.6
METs recreational and household activity	44.8	54.1	66.5	81.3	98.8	122.8
Never or former smokers,%	-	2.0	13.4	33.1	37.0	14.3
Alcohol consumption, grams/day, median						
*Men*	46.0	37.2	25.5	13.8	9.5	7.5
*Women*	24.2	18.1	11.0	4.5	2.3	1.9
***Dietary factors, grams/day, median***						
Fibre	17.1	18.3	19.6	21.4	24.0	26.3
Fruits	88.4	113.2	146.6	195.1	256.6	287.1
Vegetables	107.3	120.4	136.3	165.6	216.4	245.7
Yoghurt	4.1	8.9	16.2	24.5	40.1	53.6
Nuts	0.66	0.69	0.82	0.82	1.60	2.33
Garlic	6.9	6.8	6.6	7.7	12.6	16.0
Red and processed meat	128.0	119.0	109.5	98.9	85.5	72.7
Fish	17.5	17.5	17.9	19.7	22.8	24.0

BMI, body mass index (calculated as weight in kilograms divided by height in squared meters); EPIC, European Prospective Investigation into Cancer and Nutrition; METs, metabolic equivalents of energy expenditure (MET)-hours per week per year; SD, standard deviation.

**Table 3:** **Hazard ratios of colorectal cancer in relation to individual lifestyle factors, the EPIC cohort (1992 to 2010)**

		**Colon cancer**			**Rectal cancer**			**Colorectal cancer**		
**Healthy lifestyle factor**	**Index**	**Cases, number**	**Model 1** ^**a**^ **HR (95% CI)**	**Model 2** ^**b**^ **HR (95% CI)**	**Cases,** **number**	**Model 1** ^**a**^ **HR (95% CI)**	**Model 2** ^**b**^ **HR (95% CI)**	**Cases,** **number**	**Model 1** ^**a**^ **HR (95% CI)**	**Model 2** ^**b**^ **HR (95% CI)**
**All**										
Overweight and obesity	0	1,231	1 (Ref.)	1 (Ref.)	671	1 (Ref.)	1 (Ref.)	1,902	1 (Ref.)	1 (Ref.)
	1	1,138	0.80 (0.73-0.87)	0.80 (0.74-0.87)	719	0.93 (0.84-1.03)	0.92 (0.82-1.03)	1,857	0.84 (0.79-0.90)	0.84 (0.79-0.90)
Physical activity	0	1,144	1 (Ref.)	1 (Ref.)	648	1 (Ref.)	1 (Ref.)	1,792	1 (Ref.)	1 (Ref.)
	1	1,225	0.87 (0.80-0.95)	0.88 (0.81-0.96)	742	1.02 (0.91-1.14)	1.03 (0.92-1.15)	1,967	0.92 (0.86-0.99)	0.94 (0.87-1.00)
Smoking	0	550	1 (Ref.)	1 (Ref.)	378	1 (Ref.)	1 (Ref.)	928	1 (Ref.)	1 (Ref.)
	1	1,819	0.90 (0.82-1.00)	0.91 (0.83-1.00)	1,012	0.82 (0.72-0.93)	0.84 (0.74-0.95)	2,831	0.87 (0.81-0.94)	0.88 (0.82-0.96)
Alcohol consumption	0	695	1 (Ref.)	1 (Ref.)	462	1 (Ref.)	1 (Ref.)	1,157	1 (Ref.)	1 (Ref.)
	1	1,671	0.91 (0.83-0.99)	0.91 (0.83-1.00)	928	0.79 (0.71-0.89)	0.81 (0.72-0.91)	2,602	0.86 (0.80-0.93)	0.87 (0.81-0.94)
Diet quality	0	1,084	1 (Ref.)	1 (Ref.)	629	1 (Ref.)	1 (Ref.)	1,713	1 (Ref.)	1 (Ref.)
	1	1,285	0.86 (0.79-0.94)	0.88 (0.81-0.96)	761	0.87 (0.78-0.98)	0.89 (0.79-1.01)	2,046	0.86 (0.81-0.93)	0.88 (0.83-0.95)
**Men**										
Overweight and obesity	0	602	1 (Ref.)	1 (Ref.)	393	1 (Ref.)	1 (Ref.)	995	1 (Ref.)	1 (Ref.)
	1	427	0.73 (0.64-0.83)	0.74 (0.65-0.84)	335	0.89 (0.76-1.03)	0.90 (0.77-1.05)	762	0.79 (0.72-0.87)	0.80 (0.73-0.88)
Physical activity	0	515	1 (Ref.)	1 (Ref.)	360	1 (Ref.)	1 (Ref.)	875	1 (Ref.)	1 (Ref.)
	1	514	0.90 (0.79-1.00)	0.91 (0.80-1.03)	368	0.92 (0.79-1.08)	0.93 (0.80-1.09)	882	1.09 (0.99-1.20)	1.08 (0.98-1.19)
Smoking	0	289	1 (Ref.)	1 (Ref.)	223	1 (Ref.)	1 (Ref.)	512	1 (Ref.)	1 (Ref.)
	1	740	0.95 (0.82-1.09)	0.96 (0.83-1.10)	505	0.88 (0.75-1.04)	0.92 (0.78-1.08)	1,245	0.92 (0.83-1.02)	0.94 (0.97-1.00)
Alcohol consumption	0	377	1 (Ref.)	1 (Ref.)	287	1 (Ref.)	1 (Ref.)	664	1 (Ref.)	1 (Ref.)
	1	652	0.83 (0.73.0.95)	0.85 (0.74-0.97)	441	0.74 (0.63-0.87)	0.76 (0.64-0.89)	1,093	0.79 (0.72-0.88)	0.81 (0.73-0.89)
Diet quality	0	451	1 (Ref.)	1 (Ref.)	342	1 (Ref.)	1 (Ref.)	793	1 (Ref.)	1 (Ref.)
	1	578	0.88 (0.76-1.00)	0.89 (0.78-1.02)	386	0.78 (0.67-0.92)	0.80 (0.68-0.94)	964	0.84 (0.75-0.93)	0.85 (0.77-0.95)
**Women**										
Overweight and obesity	0	629	1 (Ref.)	1 (Ref.)	278	1 (Ref.)	1 (Ref.)	907	1 (Ref.)	1 (Ref.)
	1	711	0.85 (0.76-0.96)	0.86 (0.77-0.96)	384	0.97 (0.82-1.14)	0.95 (0.81-1.12)	1,095	0.89 (0.81-0.98)	0.89 80.81-0.97)
Physical activity	0	629	1 (Ref.)	1 (Ref.)	288	1 (Ref.)	1 (Ref.)	917	1 (Ref.)	1 (Ref.)
	1	711	0.85 (0.75-0.95)	0.86 (0.77-0.97)	374	1.16 (0.98-1.37)	1.17 (0.99-1.38)	1,085	0.94 (0.85-1.04)	0.95 (0.86-1.05)
Smoking	0	261	1 (Ref.)	1 (Ref.)	155	1 (Ref.)	1 (Ref.)	416	1 (Ref.)	1 (Ref.)
	1	1,079	0.87 (0.76-1.01)	0.88 (0.77-1.02)	507	0.76 (0.63-0.92)	0.76 (0.63-0.93)	1,586	0.83 (0.74-0.93)	0.84 (0.75-0.94)
Alcohol consumption	0	318	1 (Ref.)	1 (Ref.)	175	1 (Ref.)	1 (Ref.)	493	1 (Ref.)	1 (Ref.)
	1	1,022	0.99 (0.87-1.13)	0.99 (0.88-1.14)	487	0.90 (0.75-1.07)	0.91 (0.76-1.09)	1,509	0.96 (0.87-1.06)	0.96 (0.87-1.07)
Diet quality	0	633	1 (Ref.)	1 (Ref.)	287	1 (Ref.)	1 (Ref.)	920	1 (Ref.)	1 (Ref.)
	1	707	0.84 (0.75-0.95)	0.86 (0.77-0.97)	375	0.98 (0.83-1.17)	1.00 (0.84-1.18)	1,082	0.89 (0.81-0.98)	0.91 (0.82-1.00)

^a^Base model stratified by EPIC study centre and adjusted for age, sex, education (none, primary school, technical/professional school). ^b^Multivariable model stratified by EPIC study centre and adjusted for age, sex, education (none, primary school, technical/professional school, university degree) and after mutual adjustment for other lifestyle factors, including overweight and obesity, physical activity, smoking, alcohol consumption, and diet quality (binary variables). CI, confidence interval; EPIC, European Prospective Investigation into Cancer and Nutrition; HR, hazard ratio; PAR, population attributable fraction; Ref., reference.

**Table 4:** **Hazard ratios (HRs) of colorectal cancer according to the Healthy Lifestyle Index (HLI)** ^**a**^ **, the EPIC cohort (1992 to 2010)**

**HLI**	**Colon cancer**		**Rectal cancer**		**Colorectal cancer**	
	**Cases, number**	**HR** ^**b**^ **(95% CI)**	**Cases, number**	**HR** ^**b**^ **(95% CI)**	**Cases, number**	**HR** ^**b**^ **(95% CI)**
**All participants**						
0 or 1	216	1 (Reference)	133	1 (Reference)	349	1 (Reference)
2	526	0.85 (0.72 to 0.99)	326	0.90 (0.74 to 1.11)	852	0.87 (0.76 to 0.98)
3	816	0.78 (0.67 to 0.91)	459	0.80 (0.66 to 0.97)	1275	0.79 (0.70 to 0.89)
4	602	0.64 (0.54 to 0.75)	348	0.70 (0.57 to 0.85)	950	0.66 (0.58 to 0.75)
5	209	0.61 (0.50 to 0.74)	124	0.68 (0.53 to 0.88)	333	0.63 (0.54 to 0.74)
*P*-trend		<0.0001		<0.0001		<0.0001
Per one point increase		0.87 (0.84 to 0.91)		0.90 (0.85 to 0.94)		0.88 (0.86 to 0.92)
*P*-value^c^		<0.0001		<0.0001		<0.0001
**Men**						
0 or 1	135	1 (Reference)	94	1 (Reference)	229	1 (Reference)
2	266	0.80 (0.65 to 0.98)	196	0.87 (0.67 to 1.10)	462	0.83 (0.71 to 0.97)
3	330	0.70 (0.57 to 0.85)	236	0.75 (0.59 to 0.95)	566	0.72 (0.62 to 0.84)
4	215	0.59 (0.46 to 0.73)	160	0.66 (0.51 to 0.85)	375	0.62 (0.52 to 0.73)
5	83	0.61 (0.46 to 0.81)	42	0.47 (0.32 to 0.68)	125	0.56 (0.44 to 0.69)
*P*-trend		<0.0001		<0.0001		<0.0001
Per one point increase		0.86 (0.82 to 0.91)		0.86 (0.80 to 0.91)		0.87 (0.83 to 0.90)
*P*-value^c^		<0.0001		<0.0001		<0.0001
**Women**						
0 or 1	81	1 (Reference)	39	1 (Reference)	120	1 (Reference)
2	260	0.93 (0.73 to 1.20)	130	1.03 (0.72 to 1.48)	390	0.97 (0.78 to 1.18)
3	486	0.91 (0.71 to 1.14)	223	0.96 (0.68 to 1.36)	709	0.92 (0.76 to 1.12)
4	387	0.72 (0.56 to 0.92)	188	0.84 (0.59 to 1.19)	575	0.76 (0.62 to 0.93)
5	126	0.65 (0.48 to 0.86)	82	1.01 (0.68 to 1.49)	208	0.76 (0.60 to 0.95)
*P*-trend^c^		<0.0001		0.35		<0.0001
Per one point increase		0.88 (0.84 to 0.93)		0.97 (0.99 to 1.04)		0.91 (0.87 to 0.95)
*P*-value		<0.0001		0.35		<0.0001

^a^Healthy lifestyle index (range 0 to 5 points) is calculated by summing the binary lifestyle factor variables (0,1) including overweight and obesity, physical activity, smoking, alcohol consumption and diet quality. Participants received one point if they had any of the following behaviours: healthy weight, physically active, non-smokers or former smokers, limited alcohol consumption or healthy diet quality. ^b^Multivariable model stratified by EPIC study centre and adjusted for age at study recruitment, sex and educational status (none, primary school, technical/professional school/not specified). ^c^ *P*-value for the linear trend was calculated using the Wald test treating the index as a continuous variable. Note: *P*-interaction by sex: 0.40, for colon cancer; 0.008 for rectal cancer; 0.03 for colorectal cancer. *P* for interaction is assessed using the likelihood ratio test by generating a cross-product term between HLI and sex in the multivariable model. CI, confidence interval; EPIC, European Prospective Investigation into Cancer and Health; HR, hazard ratio.

**Table 5:** **Population attributable risks (PARs) according to individual lifestyle factors and combined Healthy Lifestyle Index (HLI)** ^**a**^ **, the EPIC Cohort (1992 to 2010)**

	**Colon cancer**		**Rectal cancer**		**Colorectal cancer**	
	**Cases, number** ^**b**^	**%PAR (95% CI)**	**Cases, number** ^**b**^	**%PAR (95% CI)**	**Cases, number** ^**b**^	**%PAR (95% CI)**
**All participants**						
*Individual lifestyle factors* ^c^ *:*						
Overweight and obesity	1,231	10 (6 to 13)	671	3 (−1 to 7)	1,902	8 (5 to 11)
Physical activity	1,144	6 (2 to 10)	648	NA	1,792	3 (0 to 6)
Smoking	550	2 (−3 to 4)	378	4 (1 to 7)	928	4 (1 to 6)
Alcohol consumption	695	2 (−1 to 4)	462	6 (2 to 9)	1,157	4 (1 to 6)
Diet quality	1,084	5 (2 to 8)	629	5 (−2 to 9)	1,713	5 (2 to 7)
***HLI <5*** ^**d**^	*2,160*	*17 (6 to 26)*	*1,266*	*13 (−4 to 27)*	*3,426*	*16 (7 to 24)*
**Men**						
*Individual lifestyle factors* ^c^ *:*						
Overweight and obesity	602	15 (8 to 21)	393	5 (−2 to 11)	995	10 (5 to 14)
Physical activity	515	4 (−2 to 9)	360	5 (−7 to 15)	875	3 (−1 to 6)
Smoking	289	1 (−2 to 4)	223	3 (−2 to 8)	512	4 (−3 to 11)
Alcohol consumption	377	5 (1 to 9)	287	12 (5 to 18)	664	7 (3 to 10)
Diet quality	451	4 (−1 to 9)	342	12 (3 to 20)	793	6 (1 to 10)
***HLI <5*** ^**d**^	*946*	*13 (−8 to 28)*	*686*	*36 (13 to 53)*	*1,632*	*22 (7 to 34)*
**Women**						
*Individual lifestyle factors* ^c^ *:*						
Overweight and obesity	629	7 (2 to 11)	278	2 (−5 to 8)	907	5 (1 to 9)
Physical activity	629	7 (1 to 12)	288	NA	917	2 (−2 to 6)
Smoking	261	11 (−27 to 37)	155	2 (−2 to 6)	416	1 (−1 to 2)
Alcohol consumption	318	NA	175	2 (−2 to 6)	493	1 (−3 to 4)
Diet quality	633	6 (1 to10)	287	NA	920	2 (0 to 4)
***HLI <5*** ^**d**^	*1,214*	*20 (6 to 32)*	*580*	*NA*	*1,794*	*11 (1 to 21)*

^a^The HLI (range 0 to 5 points) is calculated by summing the binary lifestyle factor variables (0,1) including overweight and obesity, physical activity, smoking, alcohol consumption and diet quality. Participants received one point if they had any of the following behaviours: healthy weight, physically active, non-smokers or former smokers, limited alcohol consumption or healthy diet quality. ^b^The number of cases denotes those cases without the healthy lifestyle factor or not adhering to all five healthy lifestyle factors. ^c^PAR according to each of the individual lifestyle factors, stratified by EPIC study centre, and adjusted for age, sex, education (none, primary school, technical/professional school) and mutually adjusted for the other lifestyle factors, including overweight and obesity, physical activity, smoking, alcohol consumption and diet quality (binary variables). PARs are calculated by reversing the coding of the protective factors and taking into account the relative proportion of exposed cases based on the formula from Miettinen *et al*. [31]. The 95% CIs are calculated based on the formula of Whittemore *et al*. [33]. The PARs denote the percentage of colorectal cancer cases in the population that are attributable to the non-adherence to the particular healthy lifestyle factor. ^d^PAR, stratified by EPIC study centre, and adjusted for age at study recruitment, sex and educational status (none, primary school, technical/professional school). The PAR denotes the percentage of colorectal cancer cases in the population that are attributable to the non-adherence to five healthy lifestyle behaviours. PARs are calculated based on a formula from Bruzzi *et al*. [32]. The 95% CIs are calculated based on the formula of Whittemore *et al*. [33]. Note: No meaningful PAR estimates were obtained for the associations of : physical activity and rectal cancer in all participants; alcohol consumption and colon cancer in women; as well as for physical activity and diet quality and rectal cancer in women, because the estimated hazard ratios for these individual factors in women were close to 1 (Please, see Table 3). CI, confidence interval; EPIC, European Prospective Investigation into Cancer and Nutrition.

In this large prospective cohort study over a median follow-up time of 12 years, an index based on five potentially modifiable healthy lifestyle factors including healthy weight, physical activity, non-smoking, limited alcohol consumption and a healthy diet was inversely associated with CRC risk. The associations were stronger among men compared to women, particularly for rectal cancer. If these associations were causal, 16% of the new CRC cases (22% in men and 11% in women) would have been prevented had all participants been following all five healthy lifestyles. These findings provide sex and cancer-site specific estimates of the public health burden of combined lifestyle factors for incident CRC in these European populations.

Given the high incidence and mortality rates [37], prevention strategies for reducing CRC are highly desired. In this context, there have been numerous studies exploring individual lifestyle factors with regard to CRC risk [11,38-40]. However, studies on the combined effect of lifestyle factors on CRC risk have been more sparse [41-43]. In a study of 47,927 US men in the prospective Health Professionals Follow-up Cohort, after adjusting for age and family history of CRC comparing the risk score for the combined six modifiable colon cancer risk factors (obesity, physical inactivity, alcohol consumption, early adulthood cigarette smoking, red meat consumption and low intake of folic acid from supplements) at or above the approximate 20th, 10th, or 5th percentiles versus below, the PAR% increased from 39% to 48% and 55%, respectively [41]. In the Nurses’ Health Study among 83,767 US women, those who smoked, had a consistently high relative weight, low physical activity level, consumed red or processed meat on a daily basis, were never screened, and consumed low daily amounts of folate had almost a four-fold higher risk of colon cancer by the age of 70 years [43]. Another two studies provided data for European populations. A Danish Diet Cancer and Health cohort study [42] among 55,487 men and women, reported 11% lower risk of CRC in people who adhered to five healthy lifestyle recommendations, including high physical activity, low waist circumference, not smoking, low alcohol intake and a healthy diet (dietary fibre, energy percentage from fat, red and processed meat, and fruits and vegetables). However, the study included participants only from Denmark and, therefore, its results may not be generalisable for other European populations. Using data from the EPIC cohort [44], a one-point increment in an index based on the 2007 WCRF/AICR recommendations was associated with a risk reduction of 12% (95% CI: 9% to 16%) for CRC. However, this index was based solely on BMI to define body fatness, whereas waist circumference as a measure of abdominal obesity has been suggested to be a more specific indicator for elevated metabolic risk [45]. In particular, visceral adipose tissue is physiologically more active than subcutaneous adipose tissue and generates hormones and cytokines with inflammatory, metabolic and direct carcinogenic potential, which may directly or indirectly promote cancer development. Suggested putative mechanisms that may account for the link between obesity and CRC risk include hyperinsulinaemia, chronic low-grade inflammation, altered immune response, oxidative stress, as well as disturbances in insulin-like growth factors, adipokines and sex steroids. In addition, evidence has shown that while BMI is associated with CRC risk in men only, abdominal obesity (as determined by waist circumference) is similarly strongly associated with CRC cancer both in men and in women, suggesting that it may reflect cancer risk in both sexes more adequately compared to BMI [18]. Indeed, in our data when only BMI was used to define healthy weight, the estimated HR of CRC was 0.93 (95% CI: 0.87 to 1.00), whereas the respective risk estimate for using only waist circumference was lower: HR = 0.82 (95% CI: 0.78 to 0.87). Taking the above into consideration, in our study we used both BMI and waist circumference to define healthy weight. Furthermore, the WCRF /AICR score used general dietary recommendations for cancer prevention, whereas it may be important to consider foods that have been specifically related to CRC risk. We designed an *a-priori* based healthy diet quality index that comprised individual foods specifically shown to be associated to CRC risk [2-8,11,12,38-40]. Using this index, we observed 37% lower risk of CRC for people having all five healthy lifestyle factors relative to those with none of these healthy factors.

In the present study we observed a stronger association between HLI and CRC among men than among women. Similar findings have been reported also by the Danish Diet Cancer and Health study [42], although the number of cases was much lower in that study and the interaction by sex was not statistically significant. In addition, we also observed a cancer site and sex-specific gradient in the PARs such that 36% of rectal cancer cases in men and 20% of colon cancer cases in women would have been prevented if all participants adhered to all five healthy lifestyle factors, whereas no statistically significant PARs were seen in men for colon cancer and in women for rectal cancer. These suggested sex differences could be accounted for by differences in exposure distribution among men and women, quality of reporting lifestyle data or by biological differences among sexes. Our data revealed that overweight and obesity statistically explained the association between HLI and colon cancer in men but not in women. These data are in line with previous evidence on the role of obesity as a stronger risk factor for colon cancer in men compared to women [18]. Different biological mechanisms have been suggested to explain the associations of obesity and colon cancer in men and in women. Thus, our previous work has suggested that inflammation and oxidative stress underlies this association in men, whereas hyperinsulinaemia was the candidate explaining the pathway in women [46]. More research is needed to shed light on potential biological pathways that may underlie these relations.

When interpreting PARs, it should be taken into account that these measures rely on the distribution of lifestyle factors among participants in the present cohort study. Furthermore, PARs assume that the exposures are causal and unbiased, but studies with observational design are not sufficiently able to prove this assumption. Nevertheless, this knowledge may still be useful for tailoring interventions for lifestyle modification at target population subgroups.

As lifestyle patterns occur simultaneously and the magnitude of the associations may vary according to each individual’s present factors, we also examined the associations according to different combinations of factors. In these analyses, we observed that a combination of three factors, including healthy weight, non-smoking and a healthy diet quality, was related to a lower CRC risk as low as the five factors altogether, suggesting the relative importance of this particular healthy lifestyle pattern for CRC prevention. However, due to the low prevalence of each specific combination of factors, more research is needed to investigate diversity of lifestyle patterns in relation to CRC risk.

The strengths of the present study include the large sample size, the prospective study design, and the long follow-up time of the EPIC cohort. An important advantage of the study is the availability of measured rather than self-reported anthropometric information, as well as detailed dietary and lifestyle information collected using standardised procedures and validated instruments. The present study has several limitations. In order to construct the HLI, we dichotomised each lifestyle factor according to pre-defined cut-off points. Different threshold values would have resulted in different risk estimates. The choice of cut-off points was mostly based on public health recommendations and was generalised rather than risk-specific. Because the use of equal weights is an imperfect approximation of the underlying biological relationships between the different health behaviours and CRC, future analyses should examine the potential influence of the different weightings. The dichotomisation of variables included in the lifestyle index is associated with several methodological challenges, including loss of information, power and potential for underestimating the extent of variation in risk. Discarding a high proportion of the data is particularly problematic when studies are too small and, hence, underpowered. However, EPIC is a large prospective study with a long follow-up time, therefore having sufficient power to detect underlying relationships between healthy lifestyle factors and CRC risk. Nevertheless, the likely influence of the dichotomisation of the variables in the index is underestimating the true effect of the observed associations. We used multivariable models to adjust for additional confounders; however, the potential for residual confounding remains. Measurement error in self-reported variables cannot be ruled out; however, such error would likely lead to a non-differential bias potentially leading to underestimating the true effects. We used a simplified diet quality index that may not sufficiently account for the complexity of diets. A large proportion of participants were excluded because of missing information on main exposure variables which may have potentially biased the risk estimates if the participants with missing data are not similar to those with complete data. In our data overall there have not been major differences between participants with and without missing data according to the main study characteristics and exposure variables; therefore, it is unlikely that using the complete data analysis approach would have influenced our findings. Endoscopic examinations of the large bowel have been associated with general health behaviour and lower CRC risk and, therefore, may potentially confound the association between lifestyle factors and CRC risk. Unfortunately, in the EPIC study no information on CRC screening, that is, colonoscopy or sigmoidoscopy, has been systematically collected and we were not able to account for this factor in statistical analyses. However, previous studies which controlled for colonoscopy screening did not report a change in the association between healthy lifestyle behaviours and CRC risk [47]. In addition, when we stratified the analyses by age of 50 or 55 when most screening programs in Europe are introduced, we did not observe different results; therefore, it is unlikely that the main study findings could have been largely influenced by this factor. Finally, the combined HLI did not include all possible lifestyles, that is, non-steroidal anti-inflammatory drug use or dietary nutrients (calcium, vitamin D) that could additionally influence CRC risk. If added to the index, the estimated PARs could have been potentially higher.

## Conclusions

In conclusion, combined lifestyle factors - healthy weight, high physical activity, non-smoking, limited alcohol consumption and a healthy diet - are associated with a lower CRC incidence in European populations characterized by western lifestyles. These data support the notion that the complex nature and multiple dimensions of health behaviours may be better captured in analyses of lifestyle factors in combination compared to modeling individual factors alone. From a prevention perspective, using combinations of modifiable lifestyle factors in CRC risk assessment promises to be a successful, yet simple, approach for translation of epidemiologic findings into primary cancer prevention.

## Additional files

Additional file 1: Table S1. Ethical approval review board information for local EPIC centres. Additional file 2: Table S2. Composition of the Diet Quality Index. Additional file 3: Table S3. Percent Change in Regression Coefficients for Colorectal Cancer Associated with the Healthy Lifestyle Index (continuously) with Adjustment for each Healthy Lifestyle Factor, the EPIC cohort (1992–2010). Additional file 4: Figure S1. Multivariable-adjusted Hazard Ratios of the Association between HLI and Colorectal Cancer across EPIC participating countries.

## Abbreviations

BMI: body mass index
CI: confidence interval
CRC: colorectal cancer
EPIC: European Prospective Investigation into Cancer and Nutrition
HLI: Healthy Lifestyle Index
HR: hazard ratio
PAR: population attributable risk
WCRF/AICR: World Cancer Research Fund/American Institute for Cancer Research
