# Supplementary material for: Combined impact of healthy lifestyle factors on colorectal cancer: a large European cohort study
Source: BMC Med. 2014 Oct 10;12:168. doi: 10.1186/s12916-014-0168-4 (PMC4192278; doi:10.1186/s12916-014-0168-4)
Supplement: Additional file 1: Table S1. — Ethical approval review board information for local EPIC centres. [file 12916_2014_168_MOESM1_ESM.pdf]

Table S1: Ethical approval review board information for local EPIC centres

**Center/Country**	**Ethical Review Board**
Denmark	The National Committee on Health Research Ethics
France	Comité de Protection des Personnes - France
Heidelberg, Germany	Ethics Committee of the Heidelberg University Medical School - Heidelberg, Germany
Potsdam, Germany	Ethikkommission der Landesärztekammer Brandenburg Cottbus - Potsdam, Germany
Greece	University of Athens Medical School - Athens, Greece
Milan, Italy	Comitato Etico Indipendente, Fondazione IRCCS Istituto Nazionale dei Tumori – Milano-Italy
Turn, Italy	Human Genetics Foundation Torino: Ethics Committee - Turin, Italy
The Netherlands	The Medical Ethical Committee (METC = Medisch Ethische Toetsingscommissie) of the University Medical Center Utrecht (UMCU), Utrecht - the Netherlands
Norway	Regional ethical committee for Northern Norway and the Norwegian Data Inspectorate, Norway
Spain	CEIC Comité de Ética de Investigación Clínica - Spain
Malmö, Sweden	Ethics Committee of Lundst University - Malmö, Sweden
Umea, Sweden	Umea Regional Ethical Review Board - Umea, Sweden
Cambridge, UK	Norwich District Ethics Committee - Cambridge, UK
Oxford, UK	Scotland A Research Ethics Committee (Oxford); and the Imperial College Research Ethics Committee [ICREC] (UK).
