# Supplementary material for: Combined impact of healthy lifestyle factors on colorectal cancer: a large European cohort study
Source: BMC Med. 2014 Oct 10;12:168. doi: 10.1186/s12916-014-0168-4 (PMC4192278; doi:10.1186/s12916-014-0168-4)
Supplement: Additional file 2: Table S2. — Composition of the Diet Quality Index. [file 12916_2014_168_MOESM2_ESM.pdf]

Table S2 : Composition of the Diet Quality Index

Dietary Factor		Index	Description[a]
**1**	Fibre	0	< 23.5 g/d in men and < 21.5 g/d in women
		1	≥ 23.5 g/d in men and ≥ 21.5 g/d in women
**2**	Fruits	0	< 169.4 g/d in men and < 228.3 g/d in women
		1	≥ 169.4 g/d in men and ≥228.3 g/d in women
**3**	Vegetables	0	< 160.1 g/d in men and < 185.9 g/d in women
		1	≥ 160.1 g/d in men and ≥ 185.9 g/d in women
**4**	Yoghurt	0	< 15.8 g/d in men and < 35.8 g/d in women
		1	≥ 15.8 g/d in men and ≥ 35.8 g/d in women
**5**	Nuts	0	< 0.83 g/d in men and < 0.85 g/d in women
		1	≥ 0.83 g/d in men and ≥ 0.85 g/d in women
**6**	Garlic	0	< 10.8 g/d in men and < 8.1 g/d in women
		1	≥ 10.8 g/d in men and ≥ 8.1 g/d in women
**7**	Fish	0	< 22.2 g/d in men and < 18.6 g/d in women
		1	≥ 22.2 g/d in men and ≥18.6 g/d in women
**8**	Red and processed meat	0	≥ 122.5 g/d in men and ≥ 85 g/d in women
		1	< 122.5 g/d in men and < 85 g/d in women

[a]Cut-off points are based on sex-specific median dietary intakes in non-cases (n=343,478).
