# Supplementary material for: Combined impact of healthy lifestyle factors on colorectal cancer: a large European cohort study
Source: BMC Med. 2014 Oct 10;12:168. doi: 10.1186/s12916-014-0168-4 (PMC4192278; doi:10.1186/s12916-014-0168-4)
Supplement: Additional file 3: Table S3. — Percent Change in Regression Coefficients for Colorectal Cancer Associated with the Healthy Lifestyle Index (continuously) with Adjustment for each Healthy Lifestyle Factor, the EPIC cohort (1992–2010). [file 12916_2014_168_MOESM3_ESM.pdf]

Table S3: Percent Change in Regression Coefficients for Colorectal Cancer Associated with the Healthy Lifestyle Index (continuously)[a] with Adjustment for each Healthy Lifestyle Factor, the EPIC Cohort (1992-2010)

Adjustment for individual factors:	Colon cancer	Rectal cancer	Colorectal cancer
	% Change[b] (95% CI)	% Change[b] (95% CI)	% Change[b] (95% CI)
**All participants**			
Normal weight	17 (3; 35)	-7 (-36; 17)	9 (-2; 23)
Physical activity	-2 (-19; 15)	-36 (-86; 9)	-13 (-29; 0.6)
Non-smoking	-8 (-25; 5)	16 (-8; 52)	-0.6 (-13; 12)
Limited alcohol consumption	-8 (-24; 6)	26 (1; 65)	2 (-10; 15)
Healthy diet	-0.09 (-19; 17)	2 (-30; 34)	-0.2 (-15; 15)
**Men**			
Normal weight	29 (7; 62)	-7 (-34; 17)	13 (-2; 32)
Physical activity	-0.9 (-34; 12)	-14 (-46; 8)	-11 (-29; 4)
Non-smoking	-17 (-44; 3)	-7 (-34; 17)	-13 (-30; 2)
Limited alcohol consumption	3 (-17; 26)	21 (0; 54)	11 (-4; 28)
Healthy diet	-8 (-35; 15)	10 (-16; 43)	-0.02 (-18; 18)
**Women**			
Normal weight	8 (-14; 34)	-0.03 (- 34; 35)	7 (-17; 35)
Physical activity	7 (-19; 37)	-163 (-117; 26)	-14 (-51; 13)
Non-smoking	-1 (-25; 23)	141 (21; -96)	16 (-7; 50)
Limited alcohol consumption	-20 (-50; 0)	-0.02 (-33; 31)	-13 (-42; 10)
Healthy diet	7 (-22; 40)	-0.03 (-36; 38)	3 (-29; 37)

Abbreviations: CI, confidence interval

[a]Healthy lifestyle index (range 0-5 points) is calculated by summing the binary lifestyle factor variables (0,1) including overweight and obesity, physical activity, smoking, alcohol consumption, and diet quality. Participants received 1 point if they had any of the following behaviors: normal weight, physically active, non-smokers or former smokers, limited alcohol consumption, or healthy diet quality.

[b]The percent change in the regression coefficient with adjustment for each individual lifestyle factor compared with the multivariable model. The multivariable model is stratified by EPIC study center and adjusted for age, sex and education (none, primary school, technical/professional school). The corresponding 95% confidence interval (CI) was calculated based on the Fieller's theorem.
