# Supplementary material for: Combined impact of healthy lifestyle factors on colorectal cancer: a large European cohort study
Source: BMC Med. 2014 Oct 10;12:168. doi: 10.1186/s12916-014-0168-4 (PMC4192278; doi:10.1186/s12916-014-0168-4)
Supplement: Additional file 4: Figure S1. — Multivariable-adjusted Hazard Ratios of the Association between HLI and Colorectal Cancer across EPIC participating countries. [file 12916_2014_168_MOESM4_ESM.pptx]

## Slide 1
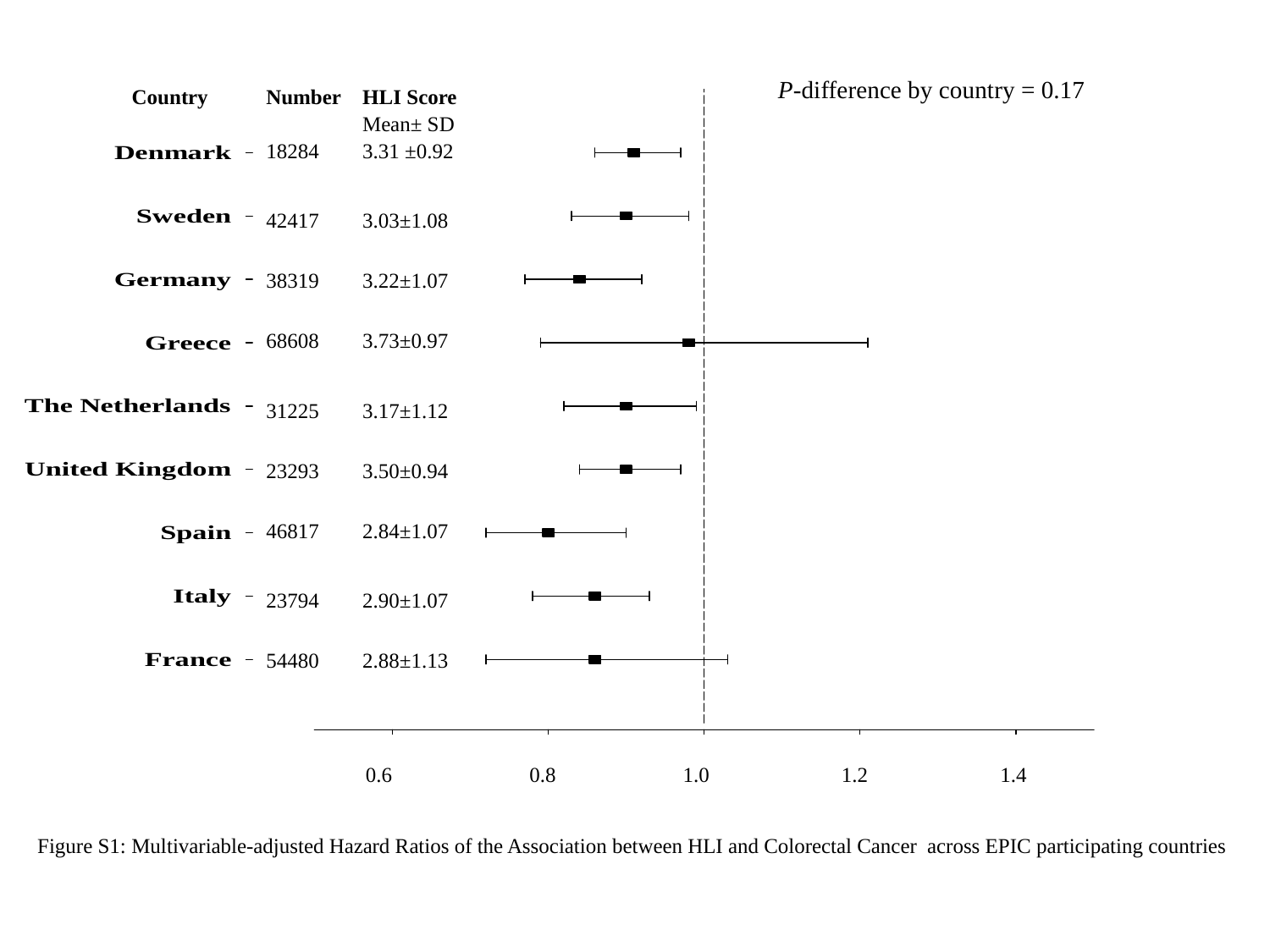

| Number | HLI Score Mean± SD |
| --- | --- |
| 18284 | 3.31 ±0.92 |
| 42417 | 3.03±1.08 |
| 38319 | 3.22±1.07 |
| 68608 | 3.73±0.97 |
| 31225 | 3.17±1.12 |
| 23293 | 3.50±0.94 |
| 46817 | 2.84±1.07 |
| 23794 | 2.90±1.07 |
| 54480 | 2.88±1.13 |
Country
P-difference by country = 0.17
 0.6 0.8 1.0 1.2 1.4
Figure S1: Multivariable-adjusted Hazard Ratios of the Association between HLI and Colorectal Cancer across EPIC participating countries
